# A rare case of spontaneous pneumothorax, pneumomediastinum and subcutaneous emphysema in the II stage of labour

**DOI:** 10.1016/j.ijscr.2020.04.011

**Published:** 2020-05-07

**Authors:** Viktor Oshovskyy, Yevheniia Poliakova

**Affiliations:** a Uniklinika Medical Centre, 4/4A Heroiv Stalinhradu str., Kyiv, Ukraine; b Multidisciplinary Clinic Of The St. Nicholas, 53 Svyatoho Mykolaya str., 69063 Zaporizhzhya, Ukraine

**Keywords:** Hamman's syndrome, Pneumomediastinum, Pneumothorax, Subcutaneous emphysema

## Abstract

**Background:**

Pneumomediastinum associated with pneumothorax and subcutaneous emphysema also known as Hamman's syndrome is a rare obstetric complication that usually happens during the second stage of labor. The causal mechanism of Hamman’s syndrome is thought to be due to excessive intrathoracic pressure associated with Valsalva maneuver which leads to distal alveolar rupture. The released alveolar air from alveolar rupture dissects along the vascular sheets down a pressure gradient toward the pulmonary hila and into the mediastinum. Prognosis is generally favorable.

**Case presentation:**

We report the case of physiologic pregnancy in a multipara parturient, which was complicated in the late second stage of labor with subcutaneous emphysema, pneumomediastinum, and severe pneumothorax. Recovery occurred after seven days of active management and a chest tube insertion. No complications were seen afterwards.

**Conclusion:**

Pneumomediastinum associated with pneumothorax and subcutaneous emphysema in the absence of an obvious precipitating cause is a rare obstetric complication and is called “Hamman’s syndrome”. The incidence of relapse is unknown, due to the number of reported cases. It could be reasonable to develop a delivery plan at 37 weeks of gestation for women with the history of Hamman syndrome. In case of tendency for a large fetus it might be wise to consider earlier induction of childbirth. Non-aggressive management of the second period of childbirth is recommended. In case of prolonged labour surgical delivery by caesarean section could be considered.

## Highlights

• Pneumomediastinum associated with pneumothorax and subcutaneous emphysema is a rare complication and is called “Hamman’s syndrome”.
• It usually happens during the second stage of labor because of the rupture of marginal alveoli into perivascular tissue planes.
• There is no specific treatment, but multidisciplinary involvement is essential.
• Prognosis is generally favorable.

## 1 Introduction

Spontaneous pneumomediastinum and subcutaneous emphysema (also known as Hamman's syndrome) is a rare obstetric complication that usually happens during the second stage of labor because of the rupture of marginal alveoli into perivascular tissue planes, with trapping of air into the mediastinum [1].

In most of cases, Hamman's syndrome is a self-limiting condition. In severe clinical presentation chest X-ray can be a useful diagnostic technique. We present the case of a 34-year-old woman with physiologic pregnancy. Her late second stage of labor was compounded be severe pneumothorax, pneumomediastinum and subcutaneous emphysema. Recovery occurred after seven days of active management and a chest tube insertion. No complications were seen afterwards.

Here we present a case of physiologic pregnancy in a multipara parturient, which was complicated in the late second stage of labor with subcutaneous emphysema, pneumomediastinum, and severe pneumothorax. The work has been reported in line with the SCARE criteria [2]

## 2 Case report

34-year-old female Gravida 5, Para 4 in her 39th week of gestation with spontaneous labor was transferred to our hospital. Her pregnancy was uncomplicated. She denied smoking and had a BMI of 24 kg/m^2^. Her past medical history was unremarkable.

The first stage of labor was uneventful and lasted for 4 h. At the end of the second stage of labor which had lasted for 30 min, the patient complained of breathlessness and right-sided chest pain. Physical examination revealed the blood pressure was 140/90 mmHg, pulse was 102/min, and the oxygen saturation was 95 % on room air oxygen. The patient was found to be moderately tachypnoeic with breath rate of 22 cycles per minute. She received oxygen supplementation by facial mask and after pushing for 10 min, the labor ended with a vaginal delivery. A healthy, live female infant (4400 g) with Apgar score of 8/9 in the first and fifth minute was delivered.

Ten minutes later she felt worse. Palpation revealed mild subcutaneous crepitus in the neck and subclavian regions corresponds to subcutaneous emphysema. In a few minutes there was marked emphysema extending from the upper anterior chest wall up to the face. The deterioration of respiratory status was observed, still the patient's condition remained stable.

**Fig. 1:**
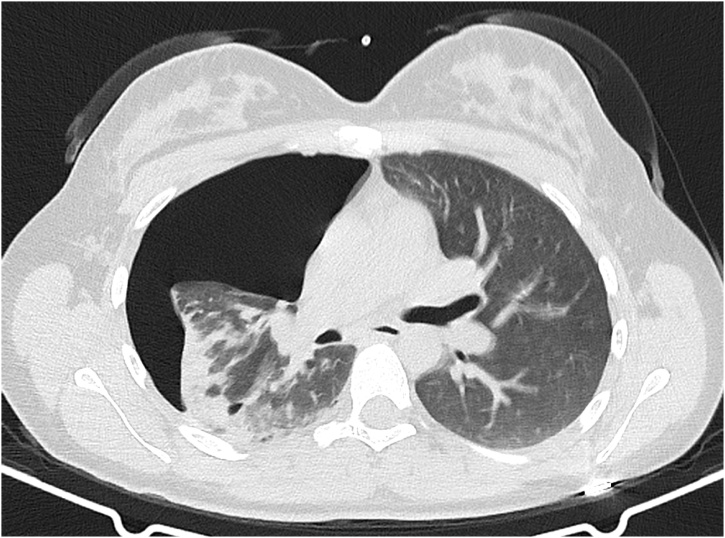
Thoracic computed tomography showing pneumothorax.

**Fig. 2:**
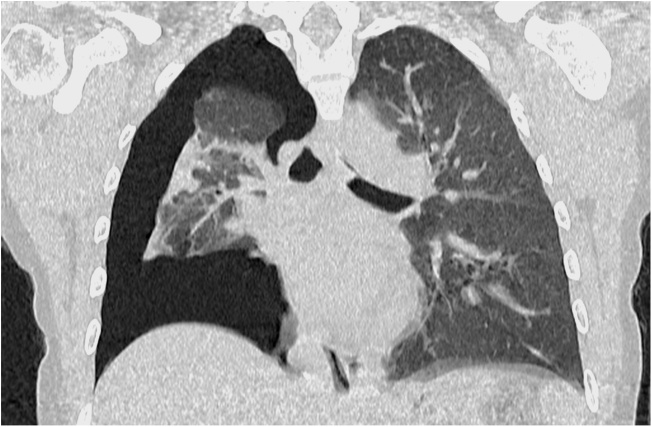
Chest CT showing large right pneumothorax with collapsed lung and pneumomediastinum.

Chest X-ray was performed which showed pneumomediastinum and pneumothorax. Computed tomography (Figs. 1, 2) confirmed right-sided tension pneumothorax, pneumomediastinum and subcutaneous emphysema. A team of thoracic surgeons were required urgently. A pigtail catheter was placed in the 5th intercostal space. The pneumothorax was resolved during few hours. The chest radiograph was repeated 5 h later which showed resolution of pneumothorax. As the clinical condition had stabilized, the chest tube was removed. The patient was observed closely for 7 days in hospital, without a specific medication with proper analgesia during the first 48 h. The symptoms regressed and repeat chest X-ray confirmed disappearance of pneumomediastinum. She was discharged home with no other postpartum problems. No complications were seen afterwards. At 2 years follow-up she is healthy and asymptomatic.

## 3 Discussion

Pneumomediastinum associated with pneumothorax and subcutaneous emphysema in the absence of an obvious precipitating cause is a rare obstetric complication. It is called “Hamman’s syndrome” and was originally described by Louis Hamman in 1939, though the first reference of this was in 1618 [3]. The prevalence is now thought to be between 1:2000 and 1:100,000 deliveries [4].

The vast majority of cases of spontaneous subcutaneous emphysema and pneumomediastinum arising during pregnancy are associated with the prolonged second stage of labor in women giving birth to large fetuses or in cases of shoulder dystocia. Severe pneumothorax is rare but possible complication. The typical sing of spontaneous pneumothorax is pleuritic chest pain associated with dyspnea [[5], [6], [7]]. Associated symptoms may include diminished breath sounds, chest expansion, tachypnea, cyanosis, tachycardia, all on the affected side. It is also may be accompanied with a crunching sound synchronous with the heartbeat, which is known as “Hamman’s sign” [8].

The causal mechanism of Hamman’s syndrome is thought to be due to excessive intrathoracic pressure associated with Valsalva maneuver which leads to distal alveolar rupture [9]. The pathophysiology was first described in 1944 by M. T. Macklin and C. C. Macklin [8,10]. The released alveolar air from alveolar rupture dissects along the vascular sheets down a pressure gradient toward the pulmonary hila and into the mediastinum [11]. This is also known as “Macklin Effect” [8]. Air may also spread through the subcutaneous tissues of the chest wall resulting in subcutaneous emphysema [12].

Prognosis: generally favorable with adequate follow-up, usually not requiring surgery, resolves spontaneously. Multidisciplinary involvement including respiratory, obstetric, anesthesiology and cardiothoracic surgery team is essential.

## 4 Conclusion

Pneumomediastinum associated with pneumothorax and subcutaneous emphysema in the absence of an obvious precipitating cause is a rare obstetric complication and is called “Hamman’s syndrome”. The incidence of relapse is unknown, due to the number of reported cases. In general, there is no tendency for recurrence in the sparing management of the II period of childbirth, however, cases of relapse in women with connective tissue dysplasia have been described.

It could be reasonable to develop a delivery plan at 37 weeks of gestation for women with a history of Hamman syndrome. In case of tendency for a large fetus it might be wise to consider earlier induction of childbirth. Non-aggressive management of the second period of childbirth is recommended. In case of prolonged labour surgical delivery by caesarean section could be considered.

## Declaration of Competing Interest

The Authors declare that there is no conflict of interest.

## Sources of funding

This research received no specific grant from any funding agency in the public, commercial or not-for-profit sectors.

## Ethical approval

Not applicable as it is a case report.

## Consent

Written informed consent was obtained from the patient for her anonymized information to be published in this article.

## Author contribution

Material preparation, data collection and analysis were performed by Oshovskyy Viktor and Poliakova Yevheniia. The first draft of the manuscript was written by Oshovskyy Viktor and all authors commented on previous versions of the manuscript. All authors read and approved the final manuscript.

Conceptualization: Oshovskyy Viktor; Poliakova Yevheniia.

Methodology: Oshovskyy Viktor.

Writing - original draft preparation: Oshovskyy Viktor.

Writing - review and editing: Poliakova Yevheniia.

Supervision: Oshovskyy Viktor.

## Registration of research studies

N/A.

## Guarantor

Oshovskyy Viktor.

## Provenance and peer review

Not commissioned, externally peer reviewed.
